# Bach1 deficiency reduces severity of osteoarthritis through upregulation of heme oxygenase-1

**DOI:** 10.1186/s13075-015-0792-1

**Published:** 2015-10-13

**Authors:** Tsuyoshi Takada, Shigeru Miyaki, Hiroyuki Ishitobi, Yuya Hirai, Tomoyuki Nakasa, Kazuhiko Igarashi, Martin K. Lotz, Mitsuo Ochi

**Affiliations:** Department of Orthopedic Surgery, Graduate School of Biomedical Sciences, Hiroshima University, 1-2-3 Kasumi, Hiroshima, 734-8551 Japan; Department of Regenerative Medicine, Hiroshima University Hospital, 1-2-3 Kasumi, Hiroshima, 734-8551 Japan; Department of Biochemistry, Tohoku University Graduate School of Medicine, 2-1 Seiryo, Aoba, Sendai, Miyagi 980-8575 Japan; Department of Molecular and Experimental Medicine, The Scripps Research Institute, 10550 N Torrey Pines Road, La Jolla, CA 92037 USA

## Abstract

**Introduction:**

BTB and CNC homology 1 (Bach1) is a transcriptional repressor of Heme oxygenase-1 (HO-1), which is cytoprotective through its antioxidant effects. The objective of this study was to define the role of Bach1 in cartilage homeostasis and osteoarthritis (OA) development using *in vitro* models and Bach1^*-/-*^ mice.

**Methods:**

HO-1 expression in Bach1^*-/-*^ mice was analyzed by real-time PCR, immunohistochemistry and immunoblotting. Knee joints from Bach1^*-/-*^ and wild-type mice with age-related OA and surgically-induced OA were evaluated by OA scoring systems. Levels of autophagy proteins and superoxide dismutase 2 (SOD2) were determined by immunohistochemistry. The relationship between HO-1 and the protective effects for OA was determined in chondrocytes treated with small interfering RNA (siRNA) targeting HO-1 gene.

**Results:**

HO-1 expression decreased with aging in articular cartilages and menisci of mouse knees. Bach1^*-/-*^ mice showed reduced severity of age-related OA and surgically-induced OA compared with wild-type mice. Microtubule-associated protein 1 light chain 3 (LC3), autophagy marker, and SOD2 were increased in articular cartilage of Bach1^*-/-*^ mice compared with wild-type mice. Interleukin-1β (IL-1β) induced a significant increase in *Adamts-5* in wild-type chondrocytes but not in Bach1^-/-^ chondrocytes. The expression of SOD2 and the suppression of apoptosis in Bach1^*-/-*^ chondrocytes were mediated by HO-1.

**Conclusions:**

Bach1 deficiency reduces the severity of OA-like changes. This may be due to maintenance of cartilage homeostasis and joint health by antioxidant effects through HO-1 and downregulation of extracellular matrix degrading enzymes. These results suggest that inactivation of Bach1 is a novel target and signaling pathway in OA prevention.

## Introduction

Osteoarthritis (OA), the most prevalent aging-related joint disease, is characterized by degradation of articular cartilage and alterations in other joint tissues. The most important risk factors are aging, obesity, mechanical stress, and inflammation, and these factors impair tissue homeostasis through dysregulation of intracellular signaling mechanisms and extracellular matrix (ECM) remodeling [1, 2]. The interaction of aging-associated changes in cartilage and molecular mechanisms of OA pathogenesis remains to be elucidated. Oxidative stress has been established as an important factor as it is elevated in joint tissues including articular cartilage in aging and OA [3–5].

Increased oxidative stress results from increased reactive oxygen species (ROS) generation and from reduced antioxidants, and is accompanied by a progressive accumulation of damaged molecules and organelles, leading to activation of catabolic factors such as inflammatory cytokines and extracellular matrix (ECM)-degrading proteases [1, 2]. Antioxidant enzymes such as Heme oxygenase-1 (HO-1) and superoxide dismutase 2 (SOD2) are an important defense against ROS-mediated damage [6, 7]. HO-1 promotes iron recycling by degrading heme into ferrous iron, carbon monoxide and biliverdin, and protects cells from various stresses [6, 8]. Previous studies revealed that induction of HO-1 has beneficial effects in several diseases [9–11]. The expression of HO-1 gene (*Hmox-1*) is negatively regulated by BTB and CNC homology 1 (Bach1), a transcriptional repressor, which binds to *Hmox-1* enhancers. Bach1 deficient mice thus have high constitutive HO-1 expression in various tissues under physiological conditions [12]. These mice have reduced disease severity in several injury models [13–15] and age-related degeneration of the meniscus [16]. However, potential beneficial effects of HO-1 in OA development have not been determined. The objectives of this study were to investigate the impact of Bach1 deficiency on two different animal models of OA, an aging model, primary OA, and a surgical model, posttraumatic OA.

## Methods

### Animal models of OA

Bach1^-/-^ mice on C57BL/6 background were described previously [12]. Only male mice were used in this study. All animal experiments were performed according to protocols approved by Hiroshima University Animal care and Use Committee. Knee joints were harvested at 6 months (n = 7), 12 months (n = 11), and 22 months (n = 14) to monitor spontaneous age-related OA. Experimental OA was induced in 10 week-old, wild-type mice (n = 13) and Bach1^-/-^ mice (n = 11) by transection of the medial meniscotibial ligament (MMTL) and the medial collateral ligament (MCL) in the right knees [17]. Mice were sacrificed 8 weeks after surgery, and the knee joints were collected for histological analysis.

### Histological assessments

All knee joints were embedded intact in paraffin after fixation in 4 % Paraformaldehyde Phosphate Buffer Solution and decalcification in K-CX (FALMA, Tokyo, Japan). Knee joints sectioned (4.5 μm) in the sagittal plane through the central weight-bearing region of the medial and lateral femorotibial joint. The sections were stained with Safranin O/fast green and at least two different sections per sample were analyzed microscopically. In this study, we applied multiple separate scoring systems for articular cartilage, meniscus, synovitis, osteophyte formation and subchondral bone thickening. Osteoarthritic damage of articular cartilage was scored using a modified Mankin system [18, 19]. Meniscus degradation was evaluated using a scoring system [20] which included the following 6 criteria, meniscus integrity (0 = smooth surface, 1 = irregularity of superficial layer or slight fibrillation, 2 = moderate fibrillation, 3 = severe fraying, tear or disruption), collagen structure (0 = normal, 1 = slight disturbance, 2 = moderate disturbance, 3 = severe disturbance or mucoid substances), cellular abnormalities (0 = normal, 1 = hypercellularity, 2 = cloning tendency, 3 = hypocellularity), stainability of safranin O staining (0 = normal, 1 = slight reduction, 2 = moderate reduction, 3 = severe reduction), calcification and cyst formation (0 = normal, 1 = slight, 2 = moderate, 3 = severe). The maximum possible score per meniscus was 18. Osteophyte formation and subchondral bone thickening were scored on a scale of 0–3, where 0 = normal, 1 = mild, 2 = moderate and 3 = severe changes, and the average scores for tibia and femur were recorded. The severity of synovitis was evaluated according to a previously described histopathological classification system [21]. The parameters of synovitis included hyperplasia/enlargement of synovial lining layer, degree of inflammatory cell infiltration and activation of resident cells/synovial stroma. All parameters were graded from 0 (absent), 1 (slight), 2 (moderate) to 3 (strongly positive) and summarized ranging from 0–9, where 0–1 corresponds to no synovitis (grade = 0), 2–3 to a slight synovitis (grade 1), 4–6 to moderate synovitis (grade 2), and 7–9 to severe synovitis (grade 3).

### Immunohistochemical analysis

Knee joint sections were immunostained with anti-HO-1 antibody (1:75, ab52947, Abcam, Austin, TX, USA) using Vectastain ABC-AP alkaline phosphatase (Vector Laboratories, Burlingame, CA, USA) as described previously [22]. For anti-microtubule-associated protein 1 light chain 3 (LC3) antibody (1:100, AP1801a, ABGENT, San Diego, CA, USA), anti-manganese superoxide dismutase (MnSOD) antibody (1:100, SPC-117, StressMarq, Victoria, BC, Canada), sections in Immunoactive pH 6.0 (Matsunami Glass, Osaka, Japan) were heated in a microwave oven and kept at 85 °C for 1.5 minutes. Slides were cooled for 20 minutes at room temperature after antigen unmasking. After washing with PBS, 3 % H_2_O_2_ treated for 10 minutes, sections were blocked with 10 % serum for 20 minutes at room temperature. Antibodies were applied and incubated overnight at 4 °C. After washing with PBS, sections were incubated with biotinylated secondary antibody for 30 minutes at room temperature and then incubated using the peroxidase based Elite ABC system (Vector Laboratories) for 30 minutes. Slides were washed, and sections were incubated with 3,3 -diaminobenzidine (DAB) substrate.

### Isolation and culture of mouse articular chondrocytes

Primary articular chondrocytes were dissected from the femoral heads of 1-month-old Bach1^-/-^ and wild-type mice by digestion with 0.3 % collagenase Type 2 (Worthington, Lakewood, NJ, USA) in Dulbecco’s modified Eagle’s medium (DMEM) (Wako, Osaka, Japan) for 2 h. Isolated chondrocytes were cultured in DMEM with 10 % fetal bovine serum.

### Transfection of small interfering RNA into mouse articular chondrocytes

Articular chondrocytes from wild-type mice and Bach1^-/-^ mice were seeded at 5 × 10^4^ cells/well on a 24-well plate and were transfected with small interfering RNA (siRNA) for HO-1 using Lipofectamine RNAiMax Reagent (Invitrogen, Carlsbad, CA, USA). The sequences of the siHO-1 were: (sense) 5′- CAACAGUGGCAGUGGGAAUTT -3′ and (antisense) 5′- AUUCCCACUGCCACUGUUGTT-3′ (Hokkaido System Sciences, Hokkaido, Japan). Control siRNAs were also prepared for the control group (siRNA negative control; siNega #1, Invitrogen). At 24 h after transfection, articular chondrocytes were treated with IL-1β (1 ng/ml; PeproTech, Rocky Hill, NJ, USA) for an additional 24 h.

### Quantitative real-time polymerase chain reaction (PCR)

Total RNA was extracted from chondrocytes using TRIzol Reagent (Invitrogen). Complementary DNA (cDNA) was synthesized using 500 ng of total RNA with the SuperScript VILO cDNA Synthesis Kit (Invitrogen). A real-time PCR assay was performed using TaqMan Gene Expression Assay probes (Applied Biosystems, Foster City, CA, USA) to amplify the *Bach1* (Mm01344527), *Hmox-1* (Mm00516005), *Col2a1* (Mm01309565_m1), *Acan* (Mm00545807), *Mmp-13* (Mm01168713), *Adamts-5* (Mm01344182_m1), and *Sod2* (Mm01313000_m1), and *Gapdh* (Mm99999915_g1) was used as the internal control to normalize the sample differences. Relative expression was calculated using the ΔΔCt values, and results were expressed as 2-ΔΔCt.

### DNA microarray analysis

DNA microarray (TORAY, Tokyo, Japan, 3D-Gene, Mouse Oligo chip 24 k) analysis was performed using total RNA from chondrocytes from wild-type mice and Bach1^-/-^ mice.

### Immunoblotting assay

For immunoblotting, proteins were extracted from cultured chondrocytes using M-PER^TM^ protein extraction reagent including protease inhibitor cocktail (Thermo Fisher Scientific, Waltham, MA, USA). Anti-HO-1 antibody (diluted 1:2000), anti-LC3 antibody (diluted 1:1000), and anti-MnSOD antibody (diluted 1:1000) were used as primary antibodies. Horseradish peroxidase (HRP)-conjugated goat anti-rabbit immunoglobulin G (IgG) antibody (sc-2030; Santa Cruz Biotechnology, Dallas, TX, USA) and anti-mouse IgG antibody (sc-2005; Santa Cruz Biotechnology) were used as secondary antibody. The signal was detected with chemiluminescence of enhanced immuno-enhancer (Wako, Osaka, Japan) using the ImageQuant LAS 4000 system (GE Healthcare, Uppsala, Sweden).

### Apoptosis assay

Articular chondrocytes were seeded at 1.5 × 10^4^ cells/well on 96-well plates and transfected with siHO-1 or siRNA negative control. Articular chondrocytes were treated with tert-butyl hydroperoxide (t-BHP) (200 uM; Wako) for 5 h at 24 h after transfection. Apoptotic chondrocytes were quantitated by counting the numbers of cell nuclei stained with Cell Event Caspase-3/7 Green Detection Reagent^TM^ and NucBlue Live cell stain ReadyProbes^TM^ (Invitrogen) in three random fields on each duplicate well at a magnification of × 10 under a fluorescence microscope (BZ-9000; Keyence, Osaka, Japan).

### Statistical analysis

The data were analyzed using the Mann–Whitney *U* test, Steel or Steel–Dwass to determine statistical differences. Differences were considered statistically significant at *P* <0.05 (*) and *P* <0.01 (**).

## Results

### Expression of HO-1 in articular cartilage

**Fig. 1:**
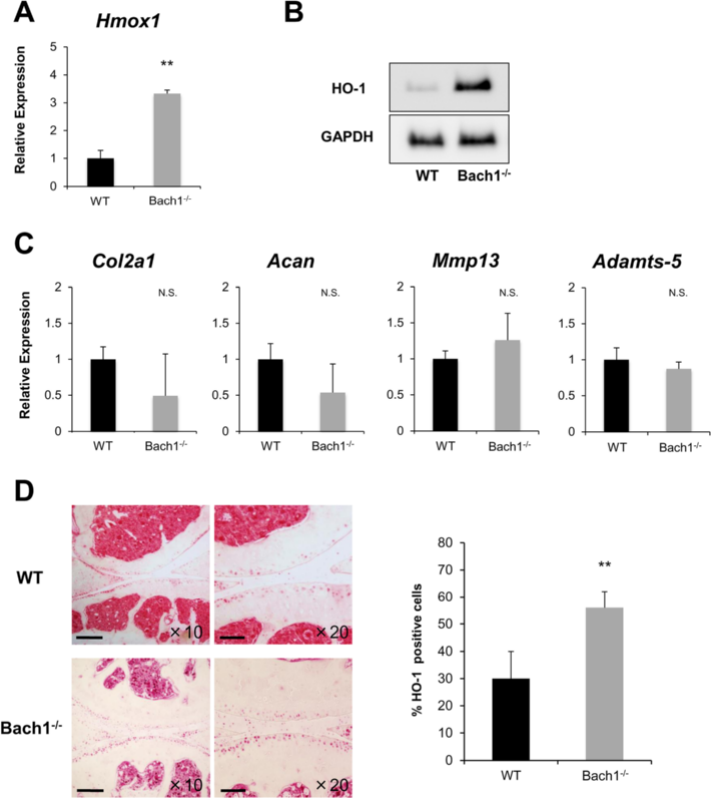
Expression of heme oxygenase-1 (*HO-1*) in articular cartilages from wild-type (*WT*) mice and Bach1^-/-^ mice. Primary articular chondrocytes were isolated from mouse femoral head cartilage (n = 4 per group) at the age of 1 month. **a** Real-time PCR assay for Hmox1. **b** Western blot analysis of HO-1 protein (n = 4). **c** Evaluation of the phenotype of articular chondrocytes in Bach1^-/-^ mice. Expression of cartilage-associated genes was determined by real-time PCR assay (n = 4 per group). **d** Comparison of HO-1 expression in articular cartilage from 22-month-old wild-type mice and Bach1^-/-^ mice (n = 4 per group). Knee joints were assessed by immunohistochemistry using anti-HO-1 antibody. Original magnification × 10 (*left*; *scale bars* 200 μm, and × 20 (*right*; *scale bars* 100 μm). Values in **a**, **c** and **d** were expressed as means ± SD. Statistical analysis was performed with the Mann–Whitney *U* test; **P <0.01

To examine the genes regulated by Bach1, DNA microarray analysis was performed on articular chondrocytes of wild-type mice and Bach1^-/-^ mice. The results showed that expression of 34 mRNAs was increased more than three-fold in Bach1^-/-^ articular chondrocytes, and only *Hmox1* was among the top 20 differentially expressed genes and highly expressed genes in Bach1^-/-^ chondrocytes (Table 1). The expression of HO-1 gene and protein were significantly increased in Bach1^-/-^ chondrocytes compared with wild-type chondrocytes (Fig. 1a, b). Studies to further evaluate the phenotype of Bach1^-/-^articular chondrocytes from 1-month-old mice observed a decrease in *Col2a1* and *Acan* with a simultaneous increase in *Mmp-13*. However, these differences were not significantly different between Bach1^-/-^ and wild-type chondrocytes (Fig. 1c), and articular cartilages from Bach1^-/-^ mice at 1 month of age did not have reduced Safranin O staining (data not shown). Furthermore, we evaluated the expression of HO-1 in knee joints from 22-month-old mice. Articular cartilage and meniscus in Bach1^*-/-*^ mice had significantly more HO-1 positive cells than wild-type mice (Fig. 1d).

**Table 1:** Gene expression in chondrocytes from Bach1^-/-^ mice

Top 20	Symbol	Ratio (Bach1^-/-^ : wild)	Top 20	Symbol	Normalized expression level
1	LOC635992	5.82	1	Fth1	23704
2	Mllt3	5.79	2	Ftl2	21177
3	4930481F22Rik	4.69	3	mt-Nd1	18945
4	Unknown	4.31	4	Fn1	18837
5	Tnfrsf26	4.29	5	unknown	18423
6	Nlrp4d	4.19	6	Rps3a	18007
7	Ly9	4.06	7	Ubb	17696
8	V1re10	4.01	8	Vim	17167
9	V1rh21	3.64	9	unknown	17069
10	**Hmox1**	**3.63**	10	Rpl35	16832
11	Lefty2	3.6	11	Gapdh	16831
12	Cdkl3	3.58	12	Col2a1	16623
13	Unknown	3.55	13	Ubb	16615
14	4933407L21Rik	3.52	14	unknown	16589
15	Arhgap4	3.5	15	**Hmox1**	**16534**
16	Serpina1f	3.43	16	EG432768	16487
17	Xmr	3.38	17	Tpt1	16335
18	Gldn	3.35	18	Ftl1	15922
19	Ckt2	3.35	19	Rpl32	15662
20	Hmgb1l	3.34	20	Ppia	15642

Total RNA was isolated from articular chondrocytes in wild-type and Bach1^-/-^ mice. Left side: top 20 of differentially expressed genes are shown as the ratio of chondrocytes in Bach1^-/-^ mice to chondrocytes in wild-type mice. Right side: the 20 genes of the highest expression level in chondrocytes from Bach1^-/-^ mice

### Expression of HO-1 in articular cartilage with aging

**Fig. 2:**
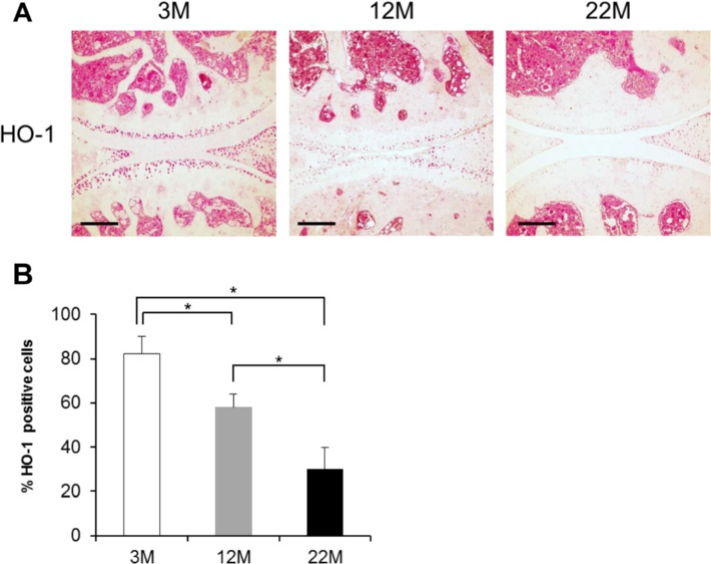
Expression of heme oxygenase-1 (*HO-1*) in knee joints in aging and osteoarthritis (OA). **a** Knee joints from 3-month- (*3 M*), 12-month (*12 M*) and 22-month (*22 M*)-old wild-type mice (n = 3–4 per group) were collected and the sections were assessed by immunohistochemistry. Original magnification × 10; *scale bars* 200 μm. **b** Values are expressed as means ± SD. Statistical analysis was performed with the Steel–Dwass test; *P <0.05

To examine aging-related changes in HO-1 expression in mouse knee joints, we performed immunohistochemical analysis. HO-1 was expressed in chondrocytes in the articular cartilage and the menisci in knee joints from wild-type mice at 3 months of age (Fig. 2a). By 12 months of age, HO-1 expression was substantially reduced to only a subset of cells in the superficial zone of cartilage (Fig. 2a). In 22-month-old mice that did not have structural cartilage defects, HO-1 staining in articular cartilage was no longer detectable, with the exception of a few cells at the joint margin (Fig. 2a). Thus, there is a significant reduction in HO-1 positive cells with aging in the articular cartilages (Fig. 2b).

### Reduced severity of osteoarthritis in Bach1^*-/-*^ mice

**Fig. 3:**
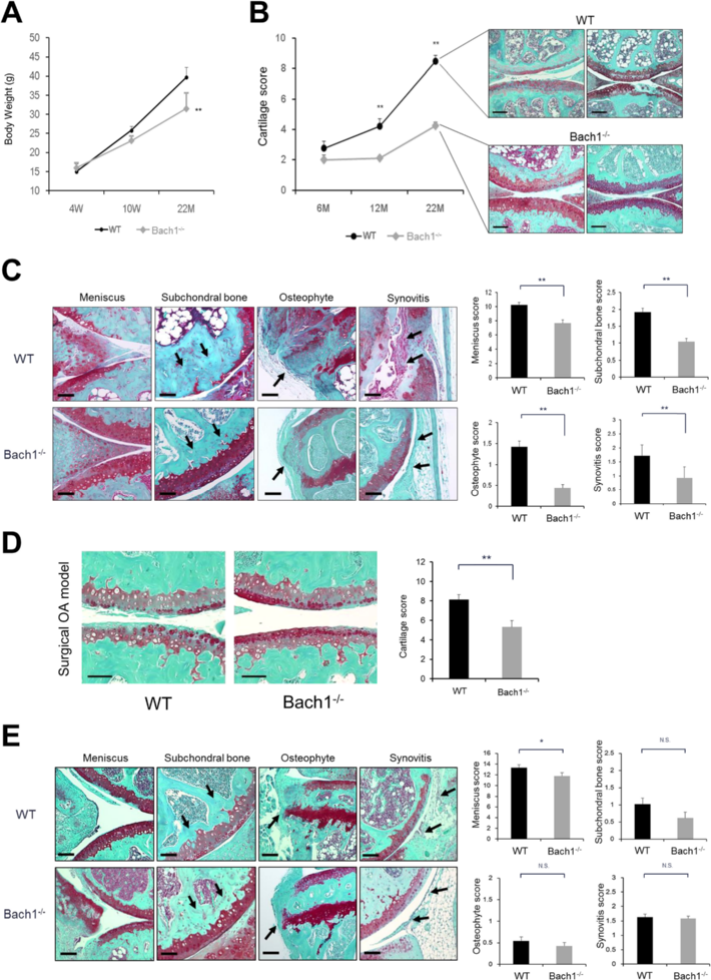
Bach1-/- mice have reduced severity of osteoarthritis (OA) in an aging model and a surgical OA model. **a** Body weight of wild-type mice and Bach1^-/-^ mice at 4 (*4 W*) and 10 weeks (*10 W*) and 22 months (*22 M*) (n = 5–9 animals per time point). **b** Histological assessment using modified Mankin scoring system of spontaneous cartilage degeneration exacerbated with aging in wild-type mice and Bach1^-/-^ mice. Photographs of a knee from a representative mouse at 22 months old, from each group are also shown. Original magnification × 10; *scale bars* 200 μm. **c** Analyses of OA pathology using the semiquantitative scoring system in mice at 22 months old, including synovitis, osteophyte formation, subchondral bone thickening and meniscus degradation. Original magnification × 20; *scale bars* 100 μm. **d** Analyses of cartilage pathology in a surgically induced OA model. Original magnification × 20; *scale bars* 100 μm. **e** Analyses of OA pathology in a surgically induced OA model, including synovitis, osteophyte formation, subchondral bone thickening and meniscus degradation. Original magnification × 20; *scale bars* 100 μm. Values are the mean ± SD. Statistical analysis was performed with the Mann–Whitney *U* test; *P <0.05, **P <0.01 versus wild-type mice

Skeletal development in Bach1^-/-^ mice was normal. There were no differences in overall joint architecture or changes in the articular cartilage and growth plate. The body weight of Bach1^-/-^ mice was not significantly different at 4 and 10 weeks, however, at 22 months it was significantly lower than in wild-type mice (Fig. 3a). To examine whether Bach1 affected age-related OA development, spontaneous osteoarthritic changes in the knee joints from Bach1^-/-^ and wild-type mice were evaluated at 6, 12 and 22 months of age. From 1 to 6 months of age, both strains of mice had intact articular cartilage and similar proteoglycan staining (data not shown). At 12 months of age, wild-type mice had a reduction in proteoglycan. OA severity in wild-type mice at 22 months of age ranged from minimal changes to cartilage fibrillation or partial and full-thickness defects with exposure of subchondral bone. These changes were significantly less severe in Bach1^-/-^ mice (Fig. 3b). Wild-type mice also exhibited meniscus degradation, inflammatory changes in synovium, osteophyte formation and subchondral bone thickening. Semiquantitative scoring systems also indicated a significant decrease in the severity of OA-like changes in all joint tissues in Bach1^-/-^ mice at 22 months of age compared with the wild-type mice (Fig. 3c). Next, we used the surgical OA model with transection of MMTL and MCL in 10-week-old mice. Consistent with observations in the aging OA model, the surgical OA model demonstrated significantly reduced articular cartilage scores in Bach1^-/-^ mice (Fig. 3d). Although the severity of meniscus degradation was accelerated by transection of the MMTL, meniscus degradation was significantly different between the wild-type and Bach1^-/-^ mice. However, inflammatory changes in synovium were not significantly different (Fig. 3e).

### SOD2 and LC3 expression in articular cartilage of aged mice

**Fig. 4:**
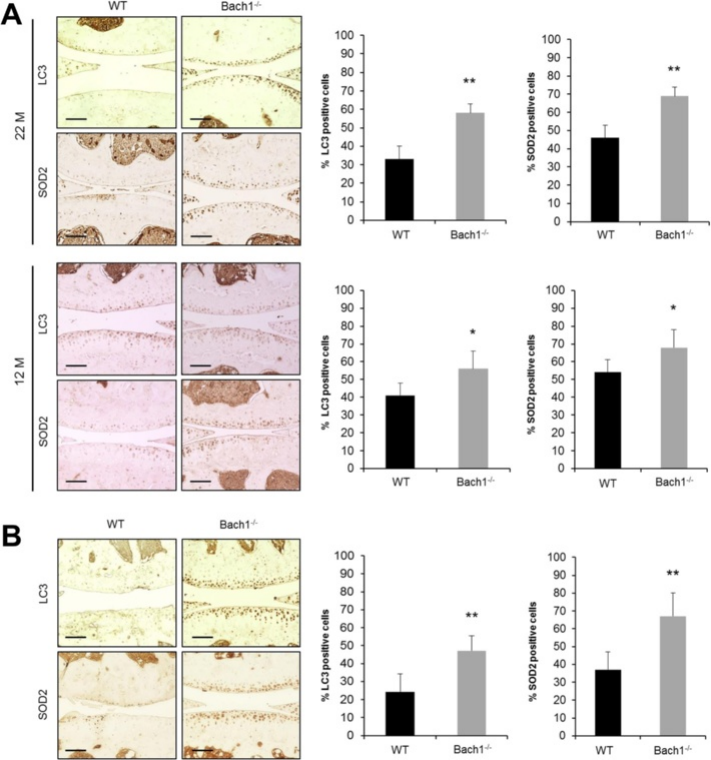
Expression of microtubule-associated protein 1 light chain 3 (*LC3*) and superoxide dismutase 2 (*SOD2*) in articular cartilage. Knee joints from wild-type (*WT*) mice and Bach1^-/-^ mice were analyzed by immunohistochemistry for LC3 and SOD2. Total number of LC3- and SOD2-positive cells in six fields were counted and the percentage of positive cells was calculated. **a** Representative immunostaining of articular cartilage from 12-month- (*12 M*) and 22 month (*22 M*)-old mice (aging model) (n = 4 per group). **b** Representative immunostaining of articular cartilage from a surgically induced osteoarthritis (*OA*) model (n = 4 per group). Original magnification × 20; *scale bars* 100 μm. Values are the mean ± SD. Statistical analysis was performed with the Mann–Whitney *U* test; *P <0.05, **P <0.01 versus wild-type mice

SOD2 and autophagy are essential cellular homeostasis mechanisms and protect against aging-related diseases [23]. To determine whether the increase in HO-1-positive cells in articular cartilage was associated with changes in SOD2 expression and autophagy, the expression of SOD2 and LC3, a main marker of autophagy, was characterized by immunohistochemistry. At 12 and 22 months of age, Bach1^-/-^ mice showed significantly increased numbers of SOD2- and LC3-positive chondrocytes compared with wild-type mice (Fig. 4a). Bach1^-/-^ mice also showed increased numbers of SOD2- and LC3-positive cells as compared to wild-type mice in the surgical OA model (Fig. 4b). These results suggest that articular cartilage in Bach1^*-/-*^ mice maintained not only HO-1 but also expression of SOD2 and the autophagy marker LC3 in the surgical and the aging-related OA model.

### The role of HO-1 and Bach1 in SOD2 and LC3 expression in mouse chondrocytes

**Fig. 5:**
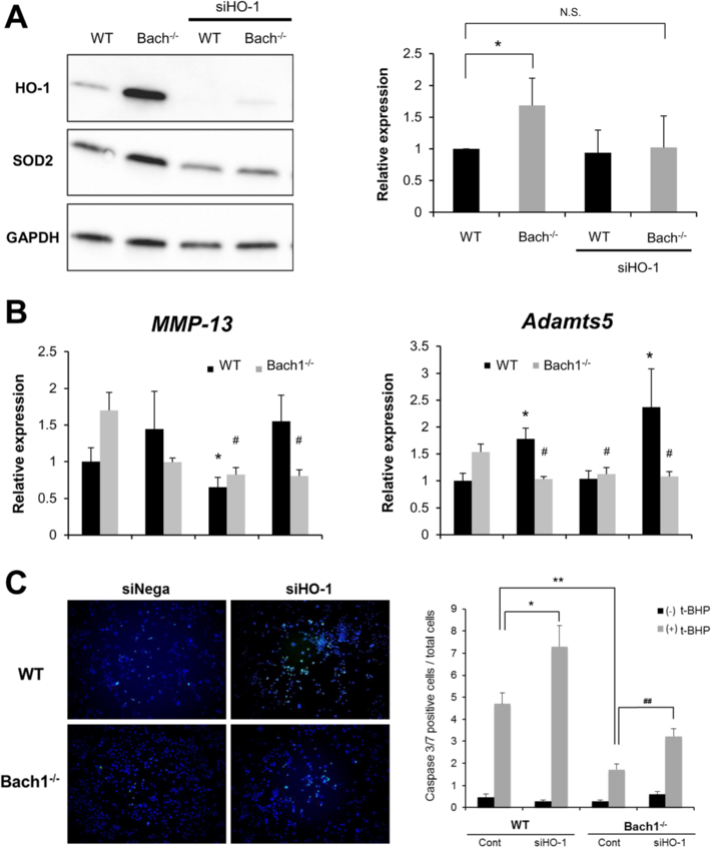
The involvement of heme oxygenase-1 (*HO-1*) in changes observed in Bach1^-/-^ articular chondrocytes. Small interfering HO-1 (*siHO-1*) or control siRNA (10 uM) was transfected into chondrocytes from wild-type (*WT*) and Bach1^-/-^ mice at 1 month of age (n = 4 per group). **a** The expression of superoxide dismutase 2 (*SOD2*) protein was detected by immunoblot analysis. Values are the mean ± SD. Statistical analysis was performed with the Steel–Dwass test; *P <0.05 versus wild-type chondrocytes. **b** The expression of *Mmp-13* and *Adamts-5* in Bach1^-/-^ chondrocytes with IL-1β (1 ng/ml) for 24 h. Values are the mean ± SD. Statistical analysis was performed with the Steel test; *P <0.05 versus wild-type chondrocytes (*-IL*); ^#^ *P* <0.05 versus Bach1^-/-^ chondrocytes (*-IL*). **c** Caspase-3/7 was detected in wild-type and Bach1^-/-^ mouse chondrocytes with or without the oxidant tert-butyl hydroperoxide (*t-BHP*) (200 uM) for 5 h. Values are the mean ± SD. Statistical analysis was performed with the Steel–Dwass test; *P <0.05, **P <0.01 versus wild-type chondrocytes with t-BHP; ^##^ *P* <0.01 versus Bach1^-/-^ chondrocytes treated with t-BHP. *GAPDH* glyceraldehyde–3–phosphate dehydrogenase

The expression of both SOD2 and LC3 increased during aging in articular cartilage of Bach1^-/-^ mice compared with wild-type mice, similar to HO-1 (Fig. 4). Thus, to elucidate the relation between Bach1, HO-1 and SOD2, LC3, articular chondrocytes from wild-type and Bach1^-/-^ mice were treated with siHO-1 or negative control siRNA. Expression SOD2 was significantly greater in chondrocytes from Bach1^-/-^ mice at 1 month of age (Fig. 5a). Furthermore, the increased SOD2 expression in Bach1^*-/-*^ mice was reduced by HO-1 knockdown (Fig. 5a). LC3 expression was not significantly different between wild-type and Bach1^-/-^ chondrocytes with or without siHO-1 (data not shown). We also examined key enzymes in OA cartilage degradation, *Mmp-13* and *Adamts-5* in IL-1β-treated Bach1^-/-^ chondrocytes. IL-1β induced a significant increase in *Adamts-5* in wild-type chondrocytes but not in Bach1^-/-^ chondrocytes. There were trends towards increased *Mmp-13* in IL-1β-treated wild-type chondrocytes but not in Bach1^-/-^ chondrocytes. Furthermore, their expression was not directly regulated by HO-1 because the knockdown of HO-1 did not significantly affect expression of these genes (Fig. 5b). Bach1^-/-^mouse chondrocytes had significantly decreased numbers of caspase-3/7-positive cells following treatment with the oxidant t-BHP. Compared with wild-type mice, the number of positive cells was significantly increased in chondrocytes treated with siHO-1 (Fig. 5c). These results suggest that upregulated SOD2 and the suppression of apoptosis in Bach1^-/-^ chondrocytes were mediated by HO-1.

## Discussion

In aging and OA, oxidative stress is elevated in joint tissues including cartilage [4]. The antioxidant enzyme HO-1 mediates general adaptive responses and provides enhanced resistance to various stresses. HO-1 is negatively regulated by Bach1, a transcriptional repressor. Bach1 is induced by transforming growth factor β (TGF-β), and is inactivated by oxidative stress, binding of heme, and oxidation of cysteine residue [12, 24–26]. HO-1 was markedly decreased in articular cartilage with aging in wild-type mice. These results suggest that maintenance of HO-1 expression has potential to protect against OA development and aging.

In the present study, Bach1^*-/-*^ mice exhibited highly increased HO-1 expression even in articular cartilage of aged mice and reduced severity of age-related OA-like changes. However, Bach1 deficiency and accompanying overexpression of HO-1 did not influence aging and life span [27]. Inflammation and obesity generally increase with aging. Our study showed that weight of Bach1^-/-^ mice were lower than wild-type mice at 22 months old. Thus, although the decreased weight of aged Bach1^-/-^ mice may contribute to joint health through metabolic changes, the level of proinflammatory cytokines in serum was not significant different between wild-type mice and Bach1^*-/-*^ mice at 22 months old (data not shown). Previously, it was reported that inflammatory diseases are less severe in Bach1^*-/-*^ mice, in part through increased HO-1 in macrophages [28]. In a similar fashion, bone destruction is attenuated in Bach1-deficient mice via altered osteoclastogenesis [29]. The induction of HO-1 also results in protective effects against inflammatory and degradative responses in OA chondrocytes and OA synoviocytes [30–32]. Thus, the reduced severity of OA-like changes in Bach1^*-/-*^ mice might occur through suppression of inflammation by higher constitutively expressed HO-1 in various cells, including chondrocytes. In aged mice and the surgical OA model, however, the level of proinflammatory cytokines in serum was not significantly different between wild-type mice and Bach1^*-/-*^ mice. In addition, the level of proinflammatory cytokines in serum from Bach1^-/-^mice was increased post-surgically compared with pre-surgical levels in the same mice (data not shown). Thus, in spite of increased proinflammatory cytokines in Bach1^-/-^ mice with surgical OA, the OA-like changes were attenuated, indicating that the reduction of OA-like changes in Bach1^*-/-*^ mice may be not only due to the anti-inflammatory effect of HO-1 upregulation.

SOD2 and autophagy also are key mediators of aging-related diseases and prevent the accumulation of defective mitochondria that produce high levels of ROS in chondrocytes [23]. SOD2 is the main antioxidant enzyme that scavenges superoxide anions in the inner mitochondrial matrix [33]. Autophagy has important anti-aging functions and reduces aging-associated cell death, dysfunction, and disease [34]. Recently, it was reported that SOD2 is reduced during OA development and in aging [5, 35], and autophagy decreased in articular cartilage of human OA, aging-related and surgically-induced OA in mice [36]. Autophagy is involved in maintenance of articular cartilage homeostasis, and reduces severity in mouse OA models [22, 37]. In the present study, Bach1^*-/-*^ mice maintained the expression of SOD2 and LC3 in articular cartilage, as well as HO-1. Although the upregulated LC3 expression in Bach1^*-/-*^ mice was not significantly reduced by HO-1 knockdown, SOD2 was regulated by HO-1. Chondrocyte-specific deletion of SOD2 in human cells and mice accelerates OA-like changes accompanied by oxidative damage and mitochondrial dysfunction [38]. Thus, maintenance of SOD2 in chondrocytes also may be important for OA prevention. While increased *Mmp-13*, *Adamts-5* and apoptosis, key OA-related factors, were suppressed in Bach1^*-/-*^ mouse chondrocytes under stress, only apoptosis was significantly regulated by HO-1. Our findings indicate that the protective effects against OA development in Bach1^*-/-*^ mice appear to be antioxidant activity and cytoprotective effects through HO-1 in chondrocytes and downregulation of ECM-degrading enzymes. Thereby, Bach1 deficiency coordinates maintenance of cartilage homeostasis and joint health.

Our observations suggest that inducers of HO-1 may be effective therapeutic agents for OA prevention. Local gene delivery of HO-1 into the mouse knee joint results in transduction of the joint tissues such as synovium, except for cartilage, however, it does not reduce OA-like changes [39]. This result suggests that the expression of HO-1 in cartilage is important for the protection and the treatment of OA. Thus, the pharmacological inhibition of Bach1 and induction of HO-1 in cartilage might have potential in the prevention or treatment of OA.

## Conclusions

Bach1^*-/-*^ mice have reduced severity of OA in aging and surgical models and this is at least in part mediated through regulation of HO-1, ECM degrading enzymes and chondrocyte viability. These findings suggest that approaches to reduce Bach1 expression have potential as a novel therapeutic approach.

## Abbreviations

Acan: aggrecan
Adamts-5: a disintegrin and metalloproteinase with thrombospondin motifs-5
Bach1: BTB and CNC homology 1
cDNA: complementary DNA
Col2a1: collagen type II alpha 1
DMEM: Dulbecco’s modified Eagle’s medium
ECM: extracellular matrix
HO-1: heme oxygenase-1
GAPDH: glyceraldehyde–3–phosphate dehydrogenase
HRP: horseradish peroxidase
IgG: immunoglobulin G
IL-1*β*: Interleukin-1*β*
LC3: microtubule-associated protein 1 light chain 3
MCL: medial collateral ligament
Mmp13: matrix metalloproteinase 13
MMTL: medial meniscotibial ligament
MnSOD: manganese superoxide dismutase
OA: osteoarthritis
PBS: phosphate-buffered saline
PCR: polymerase chain reaction
ROS: reactive oxygen species
siRNA: small interfering RNA
SOD2: superoxide dismutase 2
t-BHP: tert-butyl hydroperoxide
